# CT Hounsfield unit is a reliable parameter for screws loosening or cages subsidence in minimally invasive transforaminal lumbar interbody fusion

**DOI:** 10.1038/s41598-023-28555-7

**Published:** 2023-01-28

**Authors:** Yu-Cheng Yao, Hsien Chao, Kun-Yu Kao, Hsi-Hsien Lin, Shih-Tien Wang, Ming-Chau Chang, Chien-Lin Liu, Po-Hsin Chou

**Affiliations:** 1 Department of Orthopedics and Traumatology, Taipei Veterans General Hospital, Taipei, Taiwan; 2 School of Medicine, National Yang Ming Chiao Tung University, Taipei, Taiwan; 3 Department of Medical Education, Chang Gung Memorial Hospital, Taoyuan, Taiwan; 4 Department of Medical Education, Chi Mei Hospital, Tainan, Taiwan; 5 Kinmen Hospital, Ministry of Health and Welfare, Kinmen, Taiwan

**Keywords:** Anatomy, Diseases, Medical research, Risk factors, Signs and symptoms

## Abstract

Retrospective cohort study. To validate computed tomography (CT) radiodensity in Hounsfield units (HU) as a prognostic marker for pedicle screw loosening or cage subsidence in minimally invasive transforaminal lumbar interbody fusion (MI-TLIF). The retrospective study involved 198 patients treated with MI-TLIF. Screw loosening (SL), cage subsidence (CS), and fusion status were assessed by plain radiographs. The risk factors of SL and CS were identified using logistic regression. A total of 258 levels and 930 screws were analyzed. During a 2-year follow-up, 16.2% and 24.7% of patients had CS and SL respectively. The cut-off value of L1 HU for predicting SL or CS was 117. The L1 HU < 117 and BMI ≥ 25 were two independent risk factors. The risk of SL or CS was 4.1 fold in patients L1 HU < 117 and 2.6 fold in patients with BMI ≥ 25. For patients concurrently having BMI ≥ 25 and pre-op L1 HU < 117, the risk was 4.3 fold. Fusion rate and clinical outcome were comparable in patients with SL or CS. L1 HU < 117 and BMI > 25 were two independent risk factors that can be screened preoperatively for preventing SL or CS and lead to better management of patients undergoing MI-TLIF.

## Introduction

Lumbar degenerative disease is prevailing worldwide causing refractory low back pain and neurological symptoms and may require surgery for management^1^. Transforaminal Lumbar Interbody Fusion (TLIF) is the preferred treatment choice for degenerative lumbar disease globally^2^, which was proposed in 1998 by Harms et al.^3^. TLIF is an effective method for the management of a variety of degenerative lumbar diseases. A recent study revealed good functional outcomes and fusion rates in patients who underwent TLIF^4^. Minimally invasive-TLIF (MI-TLIF), prevents peri-operative complications such as soft tissue or muscle injury and is associated with low estimated blood loss (EBL), fewer recovery days, and allowing early ambulation to preserve patients’ muscle power^5^.

Cage subsidence and screw loosening are two common complications associated with TLIF with an incidence rate of 15–34%^6–8^ and 7–25%^9,10^ respectively. These complications may cause non-union and are associated with the clinical outcomes of back pain, leg pain, or diminished physical function^11^. Osteoporosis decreases bone strength (including bone quality and density), by altering the microarchitecture of the bone. Consequently, low bone mass may increase the complications associated with spine surgery^12^. Recent research has revealed that osteoporosis promotes complications including cage subsidence and screw loosening^7,13,14^, which significantly increases overall complications rates after TLIF^15^. Therefore, identifying patients with osteoporosis before surgery and managing it may largely reduce the incidence of surgical complications.

Dual-energy X-ray absorptiometry (DXA) is the standard tool for evaluating bone mineral density (BMD)^16^. However, DXA associated with cortical margins of vertebrae, spinal degeneration such as osteophytes, vascular calcifications, scoliosis and post-spine surgery may be misinterpreted as increased BMD^17^. DXA is expensive and carries a risk of higher radiation exposure^18^. Alternatively, studies suggest using vertebral Hounsfield Units (HU), a quantitative scale for describing radiodensity from existing CT scans to evaluate BMD without additional cost, time, or radiation exposure. HU is a proven marker for osteoporosis^19–21^. The HU reading at the L1 level is being routinely used to study patients’ BMD of the lumbar spine^19,22^. The purpose of this investigation was to determine whether preoperatively obtained HU at L1 level is a prognostic marker for predicting cage subsidence or screw loosening in MI-TLIF and other associated risk factors.

## Materials and methods

### Study design and participants

The study is based on the retrospective data collected at a single institute from October 2016 to February 2020. Institutional ethical approval was obtained before the start of this study by the Human Research Ethics Committee at Taipei Veterans General Hospital (IRB No. 2022-01-018AC). All methods were performed in accordance with the relevant guidelines and regulations, and all participants provided written, informed consent to participation.

The inclusion criteria were as follows: (1) Adult patients (2) Underwent MI-TLIF at 1- or 2- levels. (3) Followed up for more than 2 years after surgery. (4) Underwent lumbar-CT scan within 6 months before surgery. The exclusion criteria were: (1) Spinal infection, trauma, or tumor (2) Prior spine surgery (3) Patients lost to follow-up. The patients were followed up on the 1st, 3rd, 6th, 12th, and 24th months following surgery. The patients were screened using the visual analog scale score, Oswestry disability index scores, and radiographs were obtained at each follow-up visit.

### Surgical procedures

All surgeries were performed by a single senior surgeon. The patient was placed in the prone position for fluoroscopy in the anteroposterior (AP) and lateral views to locate the affected level. The surgical procedure for MI-TLIF included skin incision 2–3 cm laterally from the midline with bilateral decompression and percutaneous pedicle screw insertion. We chose the same side showing sciatica symptoms to perform facetectomy. The decompression, disc preparation, and TLIF procedures were performed under a micro-endoscopic retractor-assisted microscopic visualization. A morselized autologous bone graft accompanied with a 1-ml demineralized bone matrix (OsteoSelect® DBM Putty, Bacterin International, Inc., Belgrade, MT, USA) was used for fusion in all patients. We did not use any bone substitutes to increase the bulk of the bone grafts. We used a banana-shaped polyetheretherketone (PEEK) cage (Rainboo® lumbar cage, A-SPINE, United Orthopedic Corporation, Taiwan) in all MI-TLIF surgeries. There was no drainage tube insertion after the operation. Post-operative spinal bracing was prescribed to all patients for 3–6 months^7^.

### Data collection

All data were collected through the electronic medical record and picture archiving communication system. Patient demographics and preoperative details such as age, sex, body mass index (BMI), comorbidities, surgical time, EBL, surgical level, and hospital stay were recorded. Overweight was defined as BMI ˃ 25, as per the international classification system of the World Health Organization.

### HU evaluation

**Figure 1:**
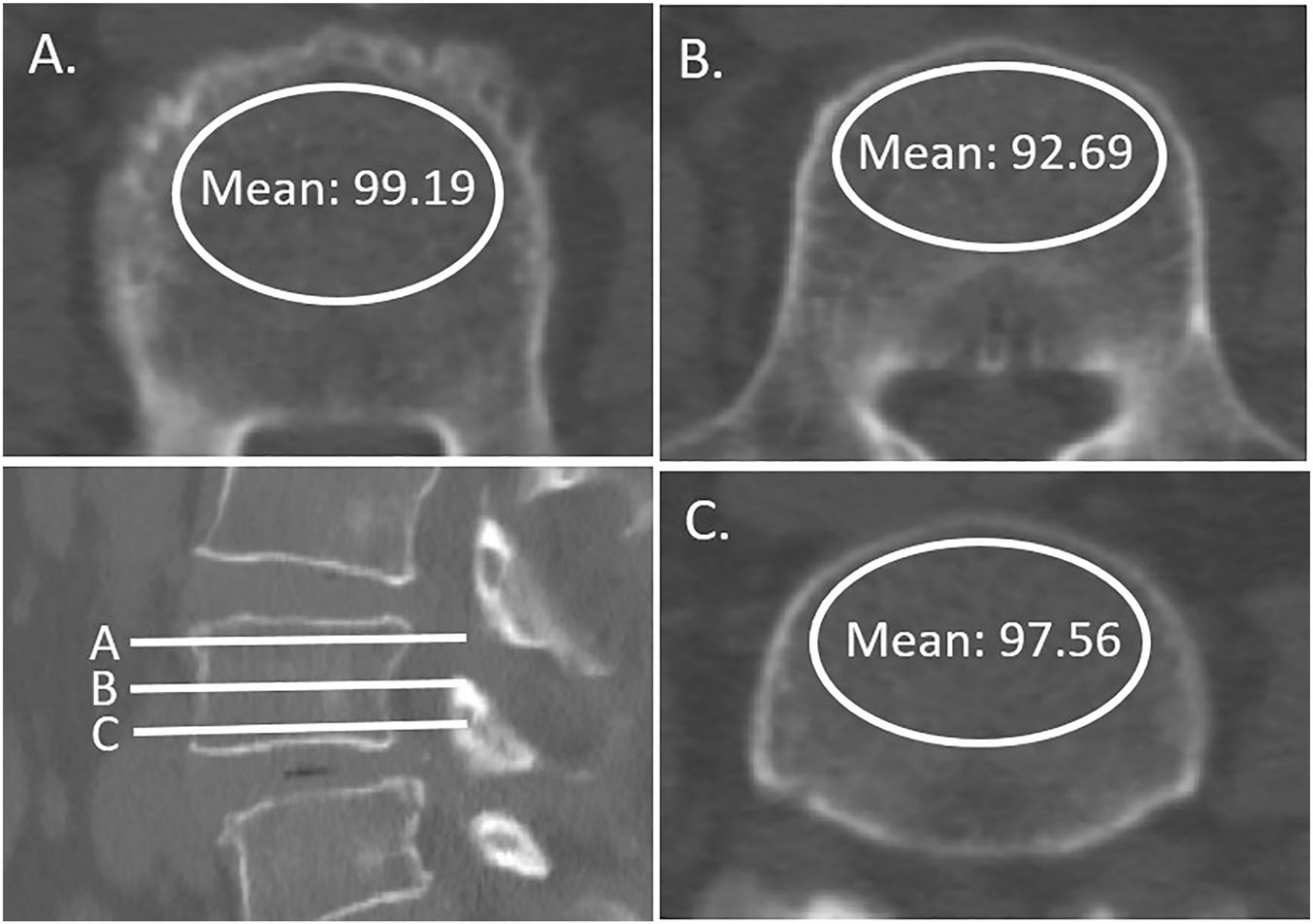
Computed tomography images illustrating the measurement of the HU value in a series of axial views of L1 vertebrae. Regions of interest were measured on the axial images at three separate locations. Slice (**A**) was taken just inferior to the superior end plate, slice (**B**) was chosen at the middle of the body, and slice (**C**) was taken just superior to the inferior end plate. Averaged HU values of the three axial slices were cut-off HU values for L1 vertebrae. *HU* hounsfield units.

A single brand of CT scanner (iCT256; Philips Brilliance) was used for all participants. CT parameters included a slice thickness of 3 mm and a tube voltage of 120 kVp. Regions of interest (ROI) were selected on the axial images of lumbar non-contrast CT at three separate locations: immediately inferior to the superior end plate, in the middle of the vertebral body, and superior to the inferior end plate (Fig. 1). For each measurement, the largest possible elliptical ROI was drawn in the vertebral body, excluding the cortical margins, lateral walls, endplates, or obvious osteophytes to prevent volume averaging. The HU values from the three axial slices were averaged to give a mean HU value for each lumbar vertebral body^19^.

### Radiographic assessment

**Figure 2:**
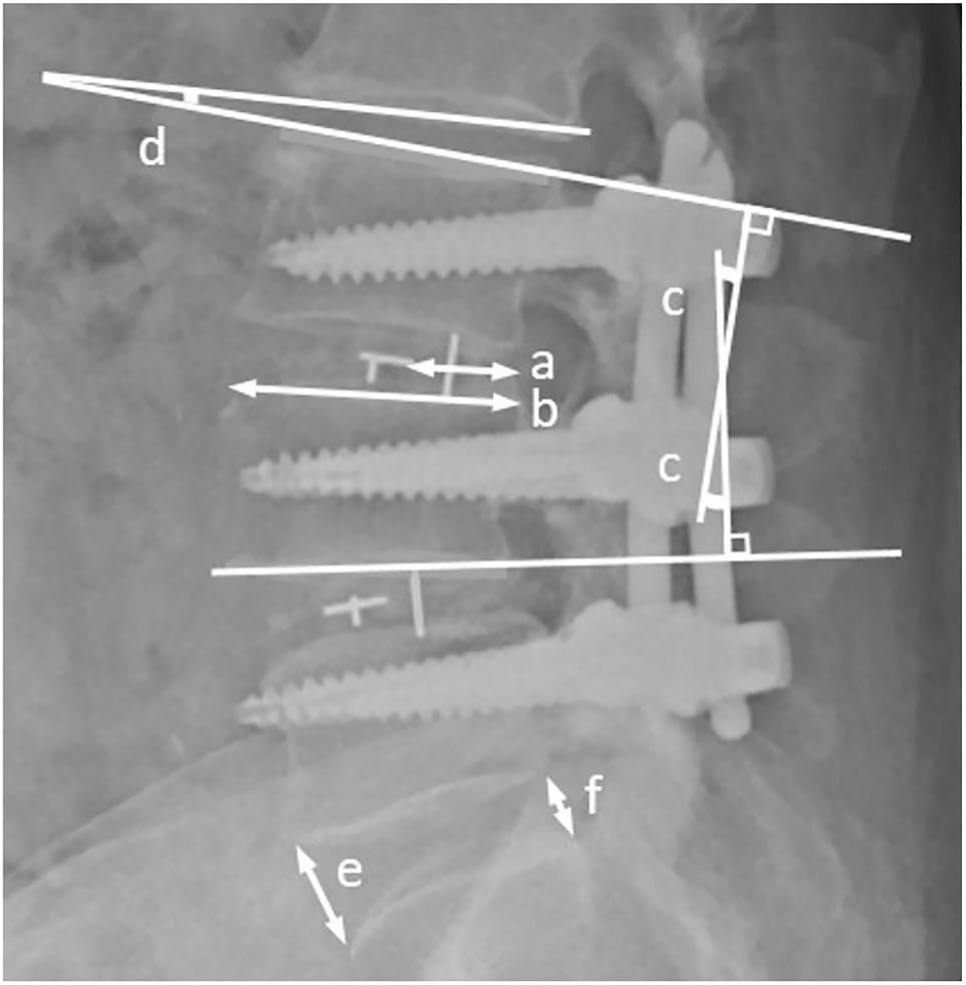
(1) The central point ratio (CPR) is the ratio of the distance between the midpoint of the cage and the posterior edge of the endplate (a); divided by the length of the superior endplate of the inferior vertebra (b). CPR = a/b × 100%. (2) Segmental lordosis angle (c) was measured at two lines parallel to the superior endplate of the cephalic surgical vertebra and the inferior end plate of the caudal surgical vertebra of the index level. (3) Disc height. Two vertical lines (e) and (f) are set at the anterior and posterior edge of the disc space, and the average of e and f was defined as the disc height. (4) Disc angle. Two lines parallel to the inferior endplate of the superior vertebra, intersect with the superior end plate of the inferior vertebra of disc space to form an angle. The angle (d) was defined as the disc angle.

**Figure 3:**
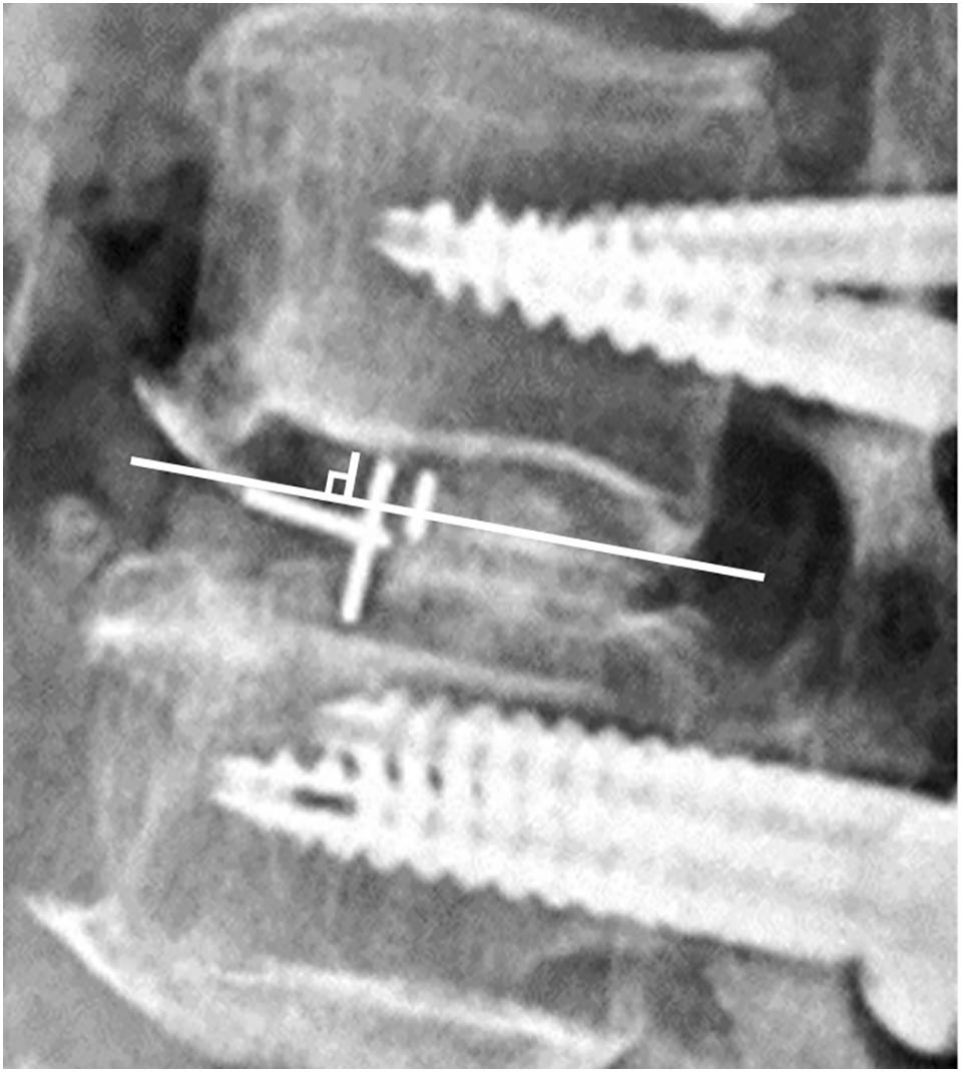
Cage subsidence was defined as a cage migrating more than 2 mm into the superior or inferior endplate of adjacent vertebrae.

**Figure 4:**
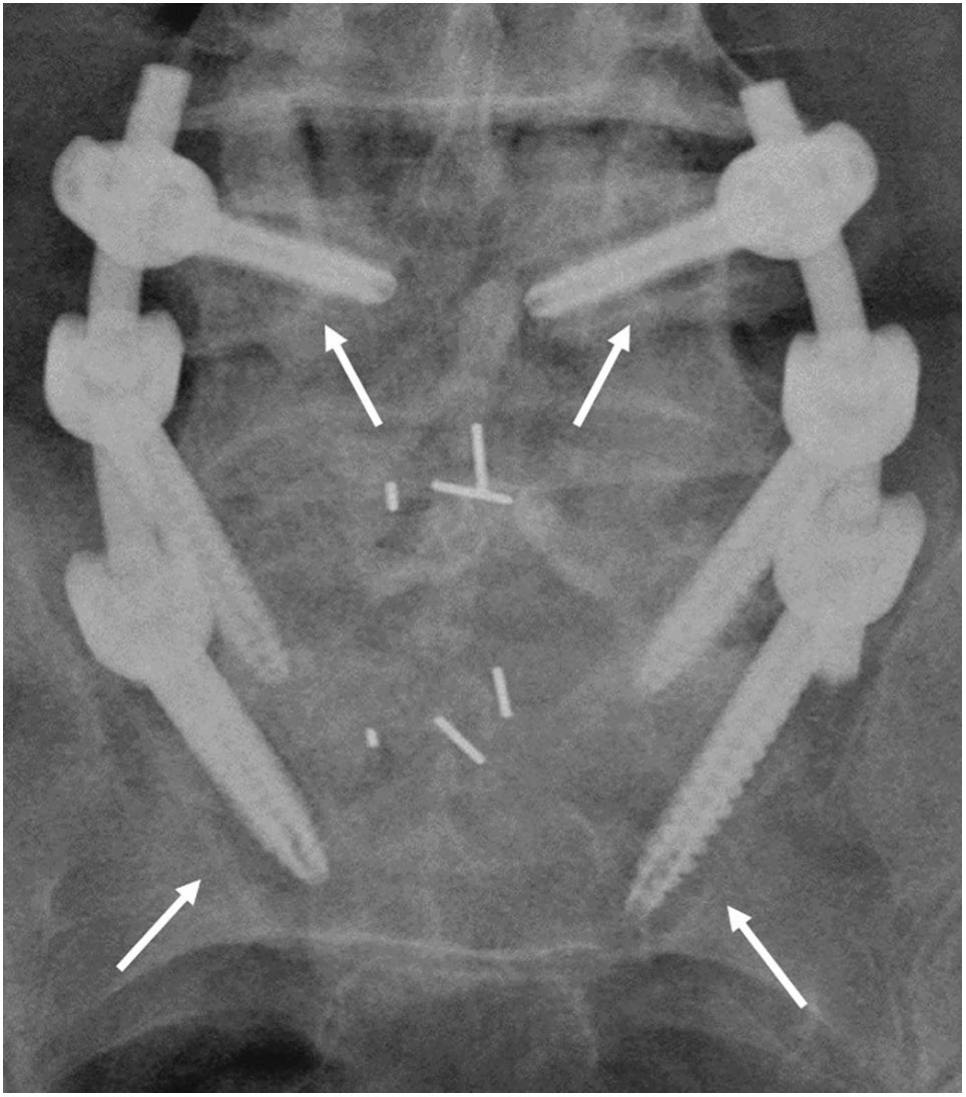
Screw loosening was defined as the presence of at least 1 mm thick radiolucent zone around any pedicle screw (Halo sign). Bilateral halo sign is evident on L3 level.

Radiographic parameters were obtained by standing anterior–posterior lateral plain radiographs. Index-level segmental lordosis was defined as the angle formed between the intersection of lines parallel to the superior endplate of the cephalic surgical vertebra and the inferior end plate of the caudal surgical vertebra on the lateral radiographs. Cage position was evaluated using a central point ratio (CPR), which is the ratio of the distance between the cage midpoint to the posterior edge of the superior endplate of the inferior vertebra (a) divided by the length of the superior endplate of the inferior vertebra (b) given by: (a/b × 100%)^7^. A CPR > 0.5 indicated a more anteriorly displaced cage. Two lines parallel to the inferior endplate of the superior vertebra and the superior end plate of the inferior vertebra of a disc space formed an intersection. The angle was defined as disc angle^23^. Disc height was measured as the average of the anterior and posterior edges of the disc space^24^ (Fig. 2). A ≥ 2 mm migration of the cage into the adjacent vertebral body was defined as cage subsidence. The migration along the superior and/or inferior end plates were labeled as settling of the implant^6^ (Fig. 3). Screw loosening was defined as the presence of at least 1 mm thick radiolucent zone around any pedicle screws^25^ (Fig. 4). The criteria for a solid fusion were defined as bone bridges with at least half of the fusion area with a minimum density as originally achieved at surgery, is considered to be mechanically solid fusion according to BSF (Brantigan, Steffee, Fraser) Criteria^26^.

### Statistical analyses

Statistical analyses were performed using IBM SPSS Statistics, Version 25.0 (IBM Corp., Armonk, NY, USA). Chi-square and Fisher exact tests were used to compare the categorical data of the two groups. Independent *t* test was used to compare continuous data. To identify the risk factors for cage subsidence or screw loosening, a univariate logistic regression (LR) analysis was performed. Variables with P ≤ 0.1 in univariate analyses were entered into a logistic regression model. The results were presented as odds ratios (OR), with 95% confidence intervals (CI). The receiver operating characteristics curve (ROC) was used to evaluate the value of HU for predicting cage subsidence and screw loosening; the area under the curve (AUC) was calculated. The two-tailed significance level was set at P < 0.05.

## Results

Our study included 198 patients with 258 levels and 930 screws. The mean follow-up period for these patients was 25.3 months. Among 198 patients, 131 were female (66.2%) and 67 were male (33.8%). The mean age of patients was 66.9 years. Most patients underwent single-level surgery (61.6%), with the most affected level being L4-L5 (44.4%) (Table 1).

**Table 1:** Demographics of the enrolled patients.

Variables	Results (n = 198)
Clinical parameters	
Age, years	66.9 ± 12.1
Sex (male/female)	67/131 (33.8%/66.2%)
Overweight	120 (60.6%)
Diabetes mellitus	48 (24.2%)
Hypertension	94 (47.5%)
Smoking	30 (15.2%)
Follow-up period, months	25.3 ± 11.0
Surgical parameters	
Level of surgery	
L34	7 (3.5%)
L45	88 (44.4%)
L5S1	27 (13.6%)
L234	3 (1.5%)
L345	46 (23.2%)
L45S1	27 (13.6%)
Total levels	
1-level	122 (61.6%)
2-level	76 (38.4%)
Surgery time, minutes	233.7 ± 71.7
EBL, mL	394.7 ± 260.7
Hospital stay, days	7.5 ± 3.7

Results are shown as mean ± standard deviation (range) or the number of patients (%).

*BMI* indicates body mass index, *BMD* bone mineral density, *HU* Hounsfield unit, *VAS* visual analog scale, *ODI* Oswestry disability index, *EBL* estimated blood loss.

### Radiographic results

During the 2-year follow-up, 32 patients had cage subsidence (16.2%), 49 patients had screw loosening (24.7%), and none of the patients encountered breaking of screws. Out of 258 cages, 34 occurrences of cage subsidence were noted, and the incidence rate of cage subsidence by the number of cages was 13.2%. Out of 930 screws that were analyzed, 100 screw loosening occurrences were noted, and the incidence rate of screw loosening by the number of screws was 10.8%.

We classified patients into two groups: patients who encountered cage subsidence or screw loosening were classified into the “CS/SL” group. Patients who did not encounter cage subsidence and screw loosening were classified into the “Non-CS/SL” group.

There were 70 patients (35.3%) in the CS/SL group.

The preoperative L1 HU was significantly lower in the CS/SL group than in the Non-CS/SL group (108.3 ± 45.3 vs. 139.8 ± 51.7, P < 0.001). There were no significant differences in age, sex, diabetes, hypertension, and smoking habits between the two groups (Table 2).

**Table 2:** Risk factors of cage subsidence or screw loosening.

Variables	Non-CS/SL (n = 128)	CS/SL (n = 70)	P value
Age, years	65.8 ± 12.6	69.0 ± 11.0	0.08
Sex (male)	49 (38.2%)	18(26.8%)	0.074
Overweight	69 (53.9%)	51 (72.9%)	**0.009**
Diabetes mellitus	31 (24.2%)	17 (24.3%)	0.992
Hypertension	57 (44.5%)	37 (52.9%)	0.262
Smoking	23 (18.0%)	7 (10.0%)	0.135
Segmental LL_preop	16.9 ± 9.6	18.0 ± 11.3	0.533
Segmental LL_postop	17.4 ± 9.7	16.7 ± 10.2	0.693
Segmental LL_delta	0.5 ± 6.6	− 1.4 ± 4.5	0.057
Disc angle_preop	6.4 ± 4.7	7.0 ± 6.0	0.505
Disc angle_postop	8.6 ± 6.2	7.6 ± 5.2	0.384
Disc angle_delta	2.2 ± 5.0	0.6 ± 4.1	0.074
Disc height_preop	0.63 ± 0.26	0.65 ± 0.20	0.784
Disc height_postop	1.02 ± 0.23	0.91 ± 0.29	0.208
Disc height_delta	0.39 ± 0.25	0.32 ± 0.25	0.133
Disc height_delta (%)	225.5 ± 1343.4	59.8 ± 58.7	0.474
Central point ratio (%)	60.9 ± 18.5	59.7 ± 10.7	0.707
Preop L1 HU	139.8 ± 51.7	108.3 ± 45.3	** < 0.001**

*LL* lumbar lordosis, *HU* Hounsfield unit, *CS* cage subsidence, *SL* screw loosening.

Significant values are in bold.

There were more overweight patients (72.9%, P = 0.009) in the CS/SL group. Cage position, disc angle, disc height and index level segmental lordosis were not significantly different between the two groups at the preoperative and postoperative stages. The cut-off value for the mean HU used to predict cage subsidence or screw loosening was 117 (sensitivity: 67.1%, specificity: 66.4%). The AUC was 0.686 (95% CI 0.608–0.765, P < 0.001).

The result of multivariate LR analysis showed that the variables ‘overweight’ and ‘L1 HU < 117’ were significant risk factors associated with cage subsidence or screw loosening. The risk of cage subsidence or screw loosening increased 2.596-fold (95% CI 1.328–5.075, P = 0.005) in overweight patients (BMI ≥ 25) and 4.100-fold in patients with lower preoperative ‘L1 HU < 117’ (95% CI 2.173–7.737, P < 0.001). Furthermore, for the patient with concurrent BMI ≥ 25 and L1 HU < 117, the OR was 4.291 with 95% CI 2.210–8.329, P < 0.001, and power = 0.991 (Table 3).

**Table 3:** Univariate logistic regression analyses and multivariate logistic regression analyses.

Variable	Univariate logistic regression analysis		Multivariate logistic regression analysis	
	OR (95% CI)	P value	aOR (95% CI)	P value
Age	1.023 (0.997–1.050)	0.083	–	–
Gender (male)	0.558 (0.293–1.062)	0.076	–	–
Overweight	2.295 (1.221–4.314)	**0.01**	2.596 (1.328–5.075)	**0.005**
Smoking	0.507 (0.206–1.250)	0.14	–	–
Preop L1 HU < 117	3.789 (2.049–7.007)	** < 0.001**	4.100 (2.173–7.737)	** < 0.001**

*HU* hounsfield units, *OR* odds ratio, *CI* confidence interval.

Significant values are in bold.

### Fusion rate and clinical outcomes

Analyses of the clinical outcomes revealed that both groups had comparable pain-scale results and functional presentation preoperatively. The Non-CS/SL-group patients had lower pain-scale results and better functional outcomes. The overall fusion rate was 82%, and a lower fusion rate was noted in CS/SC group (75.7% vs. 84.4%); however, there was no significant difference between the groups (P = 0.135). Ten patients (5.1%) underwent revision surgery due to nonunion and symptomatic screw loosening, (four patients in Non-CS/SC group [3.1%]; six patients in CS/SC group [4.4%]. There was no significant difference between the groups (P = 0.171) (Table 4).

**Table 4:** Clinical outcomes of patients between groups.

Variables	Non-CS/SL (n = 128)	CS/SL (n = 70)	P value
VAS_PreOP	6.0 ± 2.4	6.2 ± 2.7	0.599
VAS_PostOP	1.7 ± 1.8	2.6 ± 2.4	**0.021**
VAS_Delta	− 4.1 ± 2.9	− 3.5 ± 3.2	0.248
ODI_PreOP	38.9 ± 15.6	43.7 ± 22.2	0.193
ODI_PostOP	11.8 ± 12.7	19.9 ± 17.4	**0.002**
ODI_Delta	− 26.7 ± 18.6	− 22.7 ± 20.9	0.296
Fusion rate	108 (84.4%)	53 (75.7%)	0.135
Revision rate	4 (3.1%)	6 (8.6%)	0.171

*VAS* indicates visual analog scale, *ODI* Oswestry disability index, *CS* cage subsidence, *SL* screw loosening.

Significant values are in bold.

## Discussion

TLIF involving pedicle screw fixation and interbody cage placement, is a common approach for spine fusion, in the majority of cases worldwide. Cage subsidence and screw loosening are the common mechanical complications of TLIF. There are various risk factors for the occurrence of these complications; however, low BMD is the most common risk factor^7,13,14^. Our investigation confirmed that HU at the first lumbar level can be used to determine BMD and to predict the occurrence of mechanical complications associated with MI-TLIF. Being overweight is also a significant risk factor for cage subsidence or screw loosening following MI-TLIF.

A survey among spine surgeons showed that only 44% of them routinely opted for DXA examinations prior to instrumented fusion^27^. Degenerative changes in the lumbar spine can have a confounding effect by increasing the DXA scores of lumbar BMD, leading to false-negative results^28^. Alternatively, the lumbar CT is one of the routine examinations that is performed preoperatively. Spine surgeons may utilize the preoperative HU scores of the lumbar CT scan instead of DXA, to evaluate whether patients are vulnerable to screw loosening or cage subsidence following MI-TLIF.

The cage subsidence rate (16.2%) in our study was lower than that in other studies. Lin et al.^29^ showed that the cage subsidence rate was 36% after 12 months of radiograph follow-up. Yao et al.^7^ demonstrated that the cage subsidence rate was 34.1% in patients who underwent MI-TLIF surgery after 12 months of follow-up.

Studies attribute the low density of the cancellous bone to its weakened ability to resist the compression, leading to cage subsidence. Meanwhile, CT-based HU values have shown a strong correlation with BMD, as the ability of bone to withstand compression. Jie et al.^30^ reported that the mean global lumbar HU values of patients with cage subsidence were significantly low vs. controls (112.4 vs. 140.2); HU of the surgical levels in patients with cage subsidence was also significantly low vs. controls (113.4 vs. 127.9). Their cut-off values were 132 for global and 122 for the surgical region.

Barbosa et al.^31^ established the cut-off HU value of 135 at the L1 level to predict potential cage subsidence. They showed HU < 135 was an independent risk factor in cage subsidence with OR = 6 (95% CI 1.95–34) according to the multivariate analysis.

Until recently, the correlation between BMI and cage subsidence was inconclusive. Most studies predicted that higher BMI may contribute to a higher risk of cage subsidence. Behrbalk et al.^32^ reported that patients who had subsidence after lumbar interbody fusion had significantly higher BMI vs. controls (29.2 vs. 22.1). In addition, Yao et al.^7^ reported significantly higher BMI in the cage subsidence group after TLIF vs. controls (27.5 vs. 25.9). Similarly, our study demonstrated that overweight patients had a 2.596-fold higher risk of cage subsidence or screw loosening after MI-TLIF.

Previous studies have revealed that the position of the cage and the affected levels were risk factors for cage subsidence^7^. However, in our case, neither cage position nor the levels of fusion were statistically significant to be the risk factors. The contrast may be attributed to the fact that our surgeons can usually place the cage in similar positions; hence, the position of the two groups was not significantly different. There were other factors that had a much stronger impact than these two factors in cage subsidence.

The screw loosening rate in our study (24.7%) was comparable to that of other studies. For example, Ohtori et al.^9^ and Kim et al.^6^ demonstrated that the screw loosening rate was 7–25% after 12 months of follow-up for short levels of fixation; Tokuhashi et al.^33^ reported the screw loosening rate was 26.8% after 12 months follow-up in patients who underwent 1–4 levels of fixation.

Recent research has revealed that lower BMD has a detrimental effect on screw loosening. Poppenborg et al.^13^ reported that Osteoporosis, which is characterized by low BMD, was strongly associated with loosening. Bredow et al.^34^ reported screw loosening in 45 of 365 patients screened. The study revealed that the screw loosening group had a significantly lower CT-based HU vs. controls (116.3 vs. 132.7) and proposed the cut-off value of 120 HU for global HU. Schwaiger et al*.*^35^ performed a retrospective review of 38 patients who underwent CT imaging and found osteoporosis to be a significant risk factor for screw loosening in spine instrumentation. They used 117.9 HU as their cut-off which was obtained from the mean HU of L1, L2, and L3 levels, to identify patients with the risk of screw loosening. In a study involving 253 patients, Zou et al.^28^ reported a lower preoperative HU in patients with screw loosening vs. controls (106.8 vs. 129.8). The cut-off value, for identifying high-risk patients in this study was 110 HU, which was obtained from mean HU of L1 to L4 levels.

Risk factors of screw loosening have also been studied. Wu et al.^36^ reported that patients with diabetes mellitus had a significantly higher rate of screw loosening compared to controls (36.0% vs. 15.8%; p = 0.024). In addition, well-controlled serum glucose (HbA1c ≤ 8.0%) was shown to significantly reduce the chance of screw loosening (28.6% vs. 71.4%; p = 0.021). Yasushi et al.^37^ showed that high BMI was a risk factor for screw loosening after lumbosacral fixation. Studies showed that obesity may increase the mechanical stress of the posterior instrument due to the change in body shape, which could increase the screw loosening rate. These findings corresponded to our investigation.

Our study is the first to investigate risk factors of cage subsidence and screw loosening simultaneously and propose the HU cut-off to predict these complications after MI-TLIF. Low preoperative L1 HU and being overweight are indeed significant risk factors for cage subsidence and screw loosening following MI-TLIF. Our findings are novel and could promote better surgical planning. Consequently, for patients whose preoperative L1 HU < 117, pedicle screw fixation with bone cement augmentation^38^ and administration of teriparatide^9^ can be considered to prevent screw loosening. Controlling BMI by losing weight before lumbar fusion surgery also can prevent the occurrence of both cage subsidence, and screw loosening. Furthermore, endplate sclerosis at surgical levels may prevent the discovery of cage subsidence following lumbar fusion surgery^39^.

Our study has a few limitations. Firstly, we did not investigate the relationship between cage subsidence and endplate injury during surgery. Destruction of the endplate is a potential factor for cage subsidence. However, all patients were all operated on by a single surgeon, with a consistent surgical technique. We did not measure index level HU, which can be another limitation. Different scanning protocols may affect the results of HU, and we applied only one type of CT scanner in the study. Asynchronous phantom-calibrated CT may be a solution to improve accuracy and precision of measuring the BMD^40^. Nico Sollmann et al.^41^. proposed a study that HU measurments using asynchronous phantom-calibrated can have better correlation to results from QCT. However, multiple conversion formula were proposed based on different type of CT scan and parameters, we still cannot to choose a single formula to calibrate HU generally, which is a focus for future efforts.

## Conclusion

In conclusion, pre-operative L1 CT HU is a good predictor of cage subsidence or screw loosening in MI-TLIF. Patients with both a pre-op L1 HU < 117 and a BMI > 25 may be 4.29 times more likely to experience cage subsidence or screw loosening. Therefore, L1 HU < 117 and BMI > 25 were two independent risk factors that can be screened preoperatively for preventing SL or CS and lead to better management of patients undergoing MI-TLIF.

## Data Availability

The datasets used and/or analysed during the current study available from the corresponding author on reasonable request.
